# Role of interneuron subtypes in controlling trial-by-trial output variability in the neocortex

**DOI:** 10.1038/s42003-023-05231-0

**Published:** 2023-08-25

**Authors:** Lihao Guo, Arvind Kumar

**Affiliations:** 1 Division of Computational Science and Technology, School of Electrical Engineering and Computer Science, KTH Royal Institute of Technology Stockholm, Stockholm, Sweden; 2 Scilife Lab, Stockholm, Sweden

**Keywords:** Neural circuits, Neural encoding, Sensory processing, Network models

## Abstract

Trial-by-trial variability is a ubiquitous property of neuronal activity in vivo which shapes the stimulus response. Computational models have revealed how local network structure and feedforward inputs shape the trial-by-trial variability. However, the role of input statistics and different interneuron subtypes in this process is less understood. To address this, we investigate the dynamics of stimulus response in a cortical microcircuit model with one excitatory and three inhibitory interneuron populations (PV, SST, VIP). Our findings demonstrate that the balance of inputs to different neuron populations and input covariances are the primary determinants of output trial-by-trial variability. The effect of input covariances is contingent on the input balances. In general, the network exhibits smaller output trial-by-trial variability in a PV-dominated regime than in an SST-dominated regime. Importantly, our work reveals mechanisms by which output trial-by-trial variability can be controlled in a context, state, and task-dependent manner.

## Abstract (web summary)

The computational model illustrates the process by which variability in input, observed on a trial-by-trial basis, is transformed by the neural network into corresponding output variability. This showcases how distinct interneuron types contribute to variability depending on their network connectivity and context.

## Introduction

Trial-by-trial variability is a ubiquitous feature of cortical activity in vivo^1,2^. Instead of being just noise, trial-by-trial variability varies (typically gets reduced) during the stimulus presentation^2,3^, due to attentional shifts^4^ or external stimulation^5^. The presence and change in trial-by-trial variability are not merely a statistical property of neuronal activity as it is necessary for behavior^6^ and affects the stimulus-response^1^ and behavioral performance^7,8^. For example, reduction in the trial-by-trial variability affects the psychometric function^7^ and information transfer between brain regions^8^. Given the noisy inputs, stochastic neurons^9,10^, random connectivity^11,12^, and unreliable synapses^13^, trial-by-trial variability is not surprising. However, the modulation of trial-by-trial variability is usually attributed to local network structure. Computational studies have suggested that the modulation of trial-by-trial variability, particularly the stimulus-induced decrease, can be attributed to the recurrent connectivity in the local network^14–17^ and spike level correlations in the feedforward input^18^. Network models have also suggested that in a network with excitation and inhibition in balance, a reduction in the variability of excitatory neurons may be accompanied by a corresponding increase in the variability of inhibitory neurons^4^. Thus, excitatory-inhibitory interactions are crucial for the control of variability.

Previous theoretical works to unravel mechanisms underlying the modulation of trial-by-trial variability have focused on the network of a single excitatory (E) and single inhibitory (I) populations (E-I network)^14,15,17,18^. However, local networks in the brain are composed of multiple interneuron types^19^. These neuron types differ not only in their chemical signature but also in the neuron-type specific connectivity^20^. The interneuron diversity renders the local network with rich dynamical and computational properties which have not been observed in the E-I network^21–23^. However, it remains unclear when and how different interneurons contribute to trial-by-trial variability.

In the neocortex, different interneurons receive specific inputs and selectively inhibit pyramidal cells (PCs). For instance, PV-expressing interneurons are mainly driven by feedforward inputs, inhibiting perisomatic regions and basal dendrites of PCs, whereas SST-expressing interneurons are targeted by top-down inputs through VIP cells, inhibiting apical dendrites of PCs^24^. Moreover, each interneuron type can also be the target of specific neuromodulators. Therefore, by characterizing the contribution of different types of interneurons, we may uncover new mechanisms by which the brain can control the response variability in a context, state, and task-dependent manner.

To understand the role of interneurons in variability control, we investigated using a model of neocortical layer 2/3 consisting of one type of excitatory neuron (Exc) and three types of inhibitory interneuron (PV: parvalbumin, SST: somatostatin, and VIP: vasoactive intestinal polypeptide expressing cells). We refer to it as the EPSV network. First, we characterized the transfer-function of neurons in the EPSV network. Next, we measured trial-by-trial variability at different operative points. In particular, we focused on the input statistics: variance and covariance of input rate to different neuron types. We show that the main determinants of the trial-by-trial variability are the (1) balance of inputs to the different neuron populations and (2) covariance of the inputs. The effect of input covariance is contingent on the ratio of variances. The effect of these variables strongly depends on whether the network is operating in an SST or PV-dominated regime. In general, network connectivity provides a landscape on which input variability could be transformed into output variability by varying the neuron excitability, input connection strengths, and stimulus properties.

## Results

To characterize the role of interneurons in the control of trial-by-trial variability, we study how the trial-by-trial variability in the input is transferred to the output in a model of neocortex (the EPSV model, see Methods). To address this question, when synapses are static and the network does not show any hysteresis like phenomenon, it is sufficient to characterize the output by the steady-state response of the neurons. Therefore, we first estimated the transfer-function of a typical neuron in the network operating in an asynchronous-irregular and non-oscillatory state and then presented inputs with fixed trial-by-trial variability and co-variability. Our approach is similar to how linear-response theory is used to understand the transfer of input correlations^25–27^.

### Neuron transfer-function

**Fig. 1:**
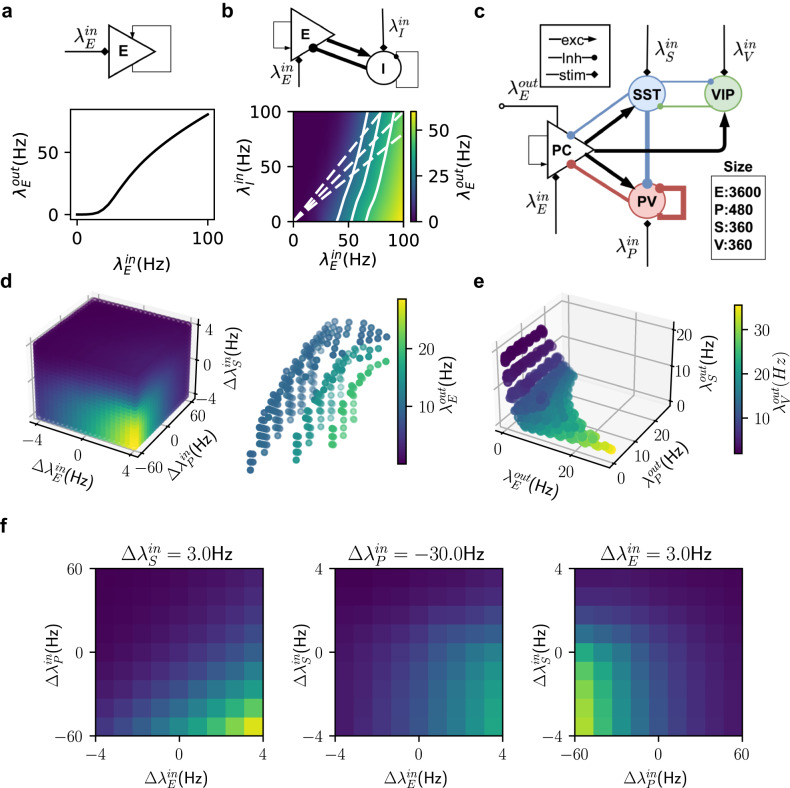
Steady-state neuron transfer-function in different networks. **a** Top Schematic of a one-population network (purely excitatory with weak recurrent connections). **a** Bottom Neuron transfer-function of the one population network where ${\lambda }_{{{{{{{{\rm{E}}}}}}}}}^{{{{{{{{\rm{in}}}}}}}}}$ denotes the rates of input (homogeneous Poisson spike trains) to excitatory neurons. **b** top Schematic of a two-population, excitatory-inhibitory (E-I), network. **b** Bottom Neuron transfer-function of the E-I network (solid white lines show constant output firing (iso-firing) rate contours and dotted white lines show inputs with different E-I ratios). **c** Schematic of the EPSV network. **d** Left transfer-function of excitatory neurons in an EPSV network zero denotes baseline input. **d** Right Three iso-firing rate surfaces with different output rates. **e** Output space of EPSV network with cubic input space as in **d**. Each dot denotes the average firing rates of four populations given certain input combination as in **d**. The three-dimensional axes shows the output activity of E, PV, and SST neurons. The color indicated the firing rate of VIP neurons. **f** 2-D slices of the transfer-function in **d** to indicate how neuron firing rate changes as a function of the inputs to two populations while the input to the third is fixed. A negative input rate denotes a reduction of input compared to the baseline (Supplementary Fig. S1).

Experimentally neuron transfer-function is typically estimated by injecting direct current at different amplitudes. However, it is more natural to inject spiking inputs. The number of different types of inputs a neuron may receive depends on the number of neuron types in the network. In a simple scenario, consider a network with only one type of excitatory neuron with weak connectivity such that the network remains stable and asynchronous. We injected Poisson-type spiking input at different rates into the network and measured the output firing rates. As expected, in this setting, the output firing rate varies monotonically as a function of the input rate (Fig. 1a). If we had injected inhibitory inputs the firing rate would have monotonically decreased from some baseline firing.

However, when a neuron is a part of a network with excitatory and inhibitory neurons (E-I network) it is important to consider both excitatory and inhibitory inputs separately to determine the neuron transfer-function^28^. This results in a two-dimensional neuron transfer-function (Fig. 1b) and a neuron can show identical output firing rate (Exc population, ${\lambda }_{{{{{{{{\rm{E}}}}}}}}}^{{{{{{{{\rm{out}}}}}}}}}$) for many different combinations of inputs to Exc and Inh populations, ${\lambda }_{{{{{{{{\rm{E}}}}}}}}}^{{{{{{{{\rm{in}}}}}}}}}$ and ${\lambda }_{I}^{{{{{{{{\rm{in}}}}}}}}}$ (shown as iso-firing rate contours: Fig. 1b, solid white lines). The neuron transfer-function also reveals how a neuron may respond if the input is varied while maintaining the ratio or balance of excitation and inhibition (Fig. 1b, dotted lines).

Extending the two-dimensional neuron transfer-function to a neuron in the EPSV circuit (Fig. 1c), we need to consider the four-dimensional input space spanned by the rate of inputs to the four populations i.e., ${\lambda }_{{{{{{{{\rm{E}}}}}}}}}^{{{{{{{{\rm{in}}}}}}}}},{\lambda }_{{{{{{{{\rm{P}}}}}}}}}^{{{{{{{{\rm{in}}}}}}}}},{\lambda }_{{{{{{{{\rm{S}}}}}}}}}^{{{{{{{{\rm{in}}}}}}}}},{\lambda }_{{{{{{{{\rm{V}}}}}}}}}^{{{{{{{{\rm{in}}}}}}}}}$ (see section Stimulus evoked input).

Because the VIP population and SST population are strongly mutually coupled to each other, we simplified by keeping ${\lambda }_{{{{{{{{\rm{V}}}}}}}}}^{{{{{{{{\rm{in}}}}}}}}}$ fixed while only changing ${\lambda }_{{{{{{{{\rm{S}}}}}}}}}^{{{{{{{{\rm{in}}}}}}}}}$ as in ref. ^22^. We estimated this three-dimensional neuron transfer-function by systematically varying the inputs (see section Stimulus evoked input) to the Exc, PV, and SST populations (Fig. 1d, left). The negative and positive inputs were relative to the baseline inputs (see section Baseline input) which were chosen to match the network activity to the in vivo firing rates of the four neuron types^29^. Rate traces and raster plots of the network working in baseline state are shown in Supplementary Fig. S1a.

As a function of the input to Exc and PV neurons, the neuron transfer-function was similar to the two-dimensional transfer-function (compare Fig. 1b (bottom) and f (left)). However, an increase in SST inputs resulted in a sharper transition compared to PV in the output of the Exc population (Fig. 1f, middle and right panels). This is because of mutual inhibitory coupling between VIP and SST neurons. Increasing the firing rate of SST neurons reduced the activity of VIP neurons which further amplified SST neurons’ firing rate and effectively silenced the PV neurons. Therefore, at some level of positive input, the network made a transition to the SST-dominated regime characterized by a sharp reduction in the activity of Exc neurons (Fig. 1f, middle and right panels).

For the three-dimensional neuron transfer-function, we can also define the manifold on which a neuron’s output remains constant despite a change in the input (Fig. 1d, right). Next, we rendered the output firing rate of all populations together to visualize all possible network states for a range of inputs to these populations. As expected, the recurrent connectivity in the network restricted the possible network states (Fig. 1e). For instance, PV and SST cells could not fire at very high levels simultaneously because of the underlying switching dynamics^22,23^. As we show in the next section, the restricted state space, the shape of iso-firing rate surfaces, and the gradient of the neuron transfer-function play a major role in determining how trial-by-trial input variability is transformed into trial-by-trial output variability.

### Trial-by-trial variability: E-I network

**Fig. 2:**
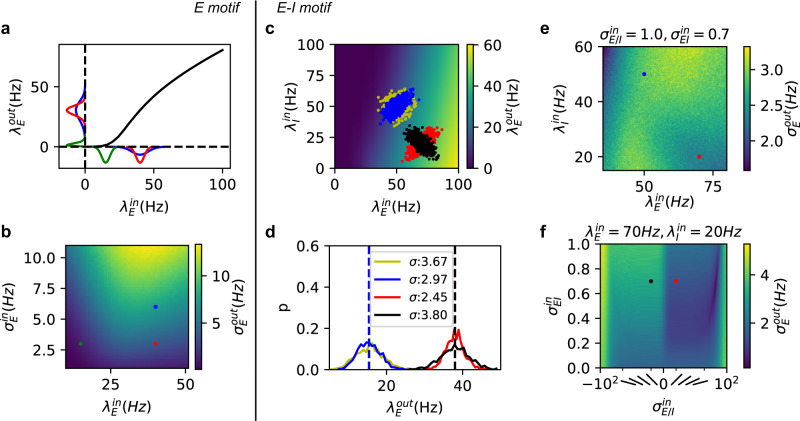
Transformation of trial-by-trial input variability to output variability. **a** A normal distribution of inputs across trials (x-axis) is transformed into a distorted distribution of outputs (*y*-axis). Different colors denote different distributions. **b** Output trial-by-trial variability (measured as standard deviation) for different mean and variance across-trial inputs in a one-dimensional case. **c** Transfer-function of the E-I network with weak recurrency. The input clouds (denoted by colors) were sampled from distinct trial-by-trial input distributions. **d** Output rate distribution of corresponding input clouds shown in **c** for the E-I network. **e** Output trial-by-trial variability as a function of mean input to Exc and Inh populations for a fixed covariance matrix. ${\sigma }_{{{{{{{{\rm{E/I}}}}}}}}}^{{{{{{{{\rm{in}}}}}}}}}$ is unitless and ${\sigma }_{{{{{{{{\rm{EI}}}}}}}}}^{{{{{{{{\rm{in}}}}}}}}}$ has unit Hz^2^. The red(blue) dot denotes the red(blue) distribution in **c**. **f** Output trial-by-trial variability as a function of covariance ${\sigma }_{{{{{{{{\rm{EI}}}}}}}}}^{{{{{{{{\rm{in}}}}}}}}}$ and balance ${\sigma }_{{{{{{{{\rm{E/I}}}}}}}}}^{{{{{{{{\rm{in}}}}}}}}}$ (slanted bars illustrate the orientation of sampling cloud as shown in **c**), for a fixed mean input to the two populations. The red(black) dot denotes the red(black) distribution in **c**.

No matter how well we control the experimental conditions, the input to a network always has some trial-by-trial variability. How this input variability is transformed into output variance depends on the gain of the neuron transfer-function in the network. If we consider a single neuron population (Fig. 2a), the task-related input follows a one-dimensional normal distribution (for simplicity) characterized by the mean *μ*^in^ and variance *σ*^in^ of the input rates across trials. For this case, output variance depends on both *μ*^in^, *σ*^in^, and the shape of the neuron transfer-function (Fig. 2a, b).

There is more freedom in controlling the trial-by-trial output variability in an E-I network. We can visualize the trial-by-trial input variability as a point cloud in a two-dimensional space spanned by inputs to the Exc and Inh populations (Fig. 2b). Each point indicates the input rate to Exc and Inh neurons in a given trial. It becomes apparent that besides the mean and the variance of the input to Exc and Inh neurons, the shape and orientation of the input point cloud are also important to determine the output variance. When the input point cloud is elongated (elliptical) and aligned to the iso-firing rate lines, output variance (contributed only by Poissonian fluctuation) will be small, irrespective of the individual input variances.

It suggests that, for the two-dimensional case, we need to consider the complete statistics of the inputs to the two populations. For simplicity, we assumed that the inputs across trials follow a bivariate normal distribution. In this setting, we need to define the following to characterize the output variance:

1 $$\vec{{\mu }^{{{{{{{{\rm{in}}}}}}}}}}=\left[\begin{array}{c}\overline{{\lambda }_{{{{{{{{\rm{E}}}}}}}}}^{{{{{{{{\rm{in}}}}}}}}}}\\ \overline{{\lambda }_{I}^{{{{{{{{\rm{in}}}}}}}}}}\end{array}\right],{\sigma }^{{{{{{{{\rm{in}}}}}}}}}=\left[\begin{array}{cc}{\sigma }_{{{{{{{{\rm{EE}}}}}}}}}^{{{{{{{{\rm{in}}}}}}}}}&{\sigma }_{{{{{{{{\rm{EI}}}}}}}}}^{{{{{{{{\rm{in}}}}}}}}}\\ {\sigma }_{IE}^{{{{{{{{\rm{in}}}}}}}}}&{\sigma }_{{{{{{{{\rm{II}}}}}}}}}^{{{{{{{{\rm{in}}}}}}}}}\end{array}\right]$$

We decomposed the distribution of the input point cloud into three factors: the trial-by-trial covariance ${\sigma }_{{{{{{{{\rm{EI}}}}}}}}}^{{{{{{{{\rm{in}}}}}}}}}={\sigma }_{IE}^{{{{{{{{\rm{in}}}}}}}}}$ (compare blue and yellow points in Fig. 2c), trial-by-trial balance ${\sigma }_{{{{{{{{\rm{E/I}}}}}}}}}^{{{{{{{{\rm{in}}}}}}}}}=\pm {\sigma }_{{{{{{{{\rm{EE}}}}}}}}}^{{{{{{{{\rm{in}}}}}}}}}/{\sigma }_{{{{{{{{\rm{II}}}}}}}}}^{{{{{{{{\rm{in}}}}}}}}}$ (compare red and black in Fig. 2c, the sign is positive when two variables are correlated and negative when two variables are anti-correlated) and the input mean $\vec{{\mu }^{{{{{{{{\rm{in}}}}}}}}}}$ (compare blue and red Fig. 2c). The three factors corresponds to three degrees of freedom which could be controlled by feed-forward connectivity as discussed in Fig. 6a. In addition, we assumed that the total input variance was fixed ${\sigma }_{{{{{{{{\rm{EE}}}}}}}}}^{{{{{{{{\rm{in}}}}}}}}}+{\sigma }_{{{{{{{{\rm{II}}}}}}}}}^{{{{{{{{\rm{in}}}}}}}}}={{{{{{{\rm{const}}}}}}}}$. All rates have unit Hz; trial-by-trial variances and covariances have unit Hz^2^; trial-by-trial balances are unitless.

First, we fixed the trial-by-trial covariance and variance of inputs to Exc and Inh populations and systematically varied the Exc and Inh input means. We used the neuron transfer-function, estimated from network response during ongoing activity state, to obtain the corresponding output rates. The trial-by-trial variability reflected the gradient of the 2-dimensional neuron transfer-function and varied greatly as we changed the input means (Fig. 2e). Typically, low firing rate regions were associated with high variability (Fig. 2e). The Fig. 2e show that in an E-I network if the input covariance matrix remains fixed, just a change in the mean input, which drives the network to a higher firing rate, is sufficient to reduce trial-by-trial variability (Fig. 2c, blue and red histogram, and Fig. 2e, blue and red dots) unless we are operating at very low evoked firing rates. This observation provides a new explanation of the reduction in the trial-by-trial variability during evoked activity^2^. However, from the Fig. 2e we can also derive constraints on Exc and Inh inputs which will increase trial-by-trial variability during evoked activity.

Next, we systematically varied covariance between inputs to Exc and Inh neurons and the balance of inputs to Exc and Inh neurons while keeping the mean of inputs constant (Fig. 2f). This analysis is consistent with the idea that output variance is minimal when the input point cloud is elliptical with a positive slope and aligned to the iso-firing rate lines. It shows that when Exc and Inh inputs are anti-correlated (Fig. 2 black distribution), the input point cloud is orthogonal to the iso-firing rate lines, output variance is maximal.

### Trial-by-trial variability: EPSV network

**Fig. 3:**
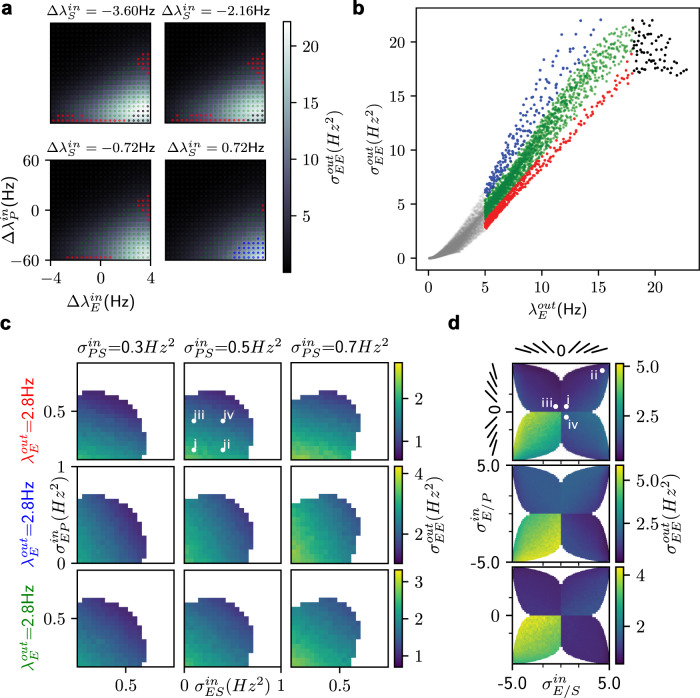
Trial-by-trial variability in EPSV circuit derived from neuron transfer-function. **a** Output trial-by-trial variability (quantified as variance) of E population as a function of across-trial input means with a fixed covariance matrix (inputs were sampled from an un-correlated tri-variate normal distribution). Colorbar shows the variance. Red, blue, and green dots correspond to the network in a PV-dominated, SST-dominated, and PV-SST regime (see Fig. S1a–c). Black dots refer to a high firing rate region where output variance decreases when increasing the output firing rate. **b** Output trial-by-trial variability as a function of output rate of E neurons. Each dot corresponds to a specific input (see the left panel). Same color code as in left panel. **c** Output trial-by-trial variability as a function of the trial-by-trial covariance of inputs with a fixed variance ${\sigma }_{{{{{{{{\rm{EE}}}}}}}}}^{{{{{{{{\rm{in}}}}}}}}}=1.8,\,{\sigma }_{{{{{{{{\rm{PP}}}}}}}}}^{{{{{{{{\rm{in}}}}}}}}}={\sigma }_{{{{{{{{\rm{SS}}}}}}}}}^{{{{{{{{\rm{in}}}}}}}}}=0.6$. From the top row to the bottom row, the network was tuned to operate in PV-dominated (red) or SST-dominated (blue), or PV-SST driven (green) regimes with similar output rates. (white regions are the cases when covariance matrix is not semi-definite positive, see Supplementary S1a-c for spiking activity rasters.) **d** Output trial-by-trial variability as a function of the trial-by-trial balance with fixed covariance ${\sigma }_{{{{{{{{\rm{PS}}}}}}}}}^{{{{{{{{\rm{in}}}}}}}}}={\sigma }_{{{{{{{{\rm{ES}}}}}}}}}^{{{{{{{{\rm{in}}}}}}}}}={\sigma }_{{{{{{{{\rm{EP}}}}}}}}}^{{{{{{{{\rm{in}}}}}}}}}=0.5{{{{{{{{\rm{Hz}}}}}}}}}^{2}$. Slanted bars indicate the orientation of the input point cloud. Roman numerals in the **c** and **d** refer to the four cases simulated in Fig. 5 and Fig. 4.

Next, we used the same approach described in the previous section to isolate the role of different interneurons in shaping trial-by-trial variability. First, we fixed the covariances and variances (assuming non-structured inputs following an uncorrelated standard tri-variate normal distribution) and systematically varied the mean of the input to different neurons. Given that network interactions render the neuron transfer-function nonlinear, the trial-by-trial variance was non-linear as a function of mean input even with uncorrelated inputs (zero covariance). (Fig. 3a). Unlike the E-I network (Fig. 2e), in the EPSV network for most cases, output variability was positively correlated with output rate (Fig. 3b). However, it was possible to find input configurations when an increase in output firing rate was associated with a decrease in trial-by-trial variability. But given trial-by-trial inputs following an uncorrelated standard tri-variate normal distribution, such a reduction was only possible for high firing rates when neurons’ transfer-function saturated (Fig. 3b black dots). Therefore, this cannot be the explanation of the reduction in trial-by-trial variability during evoked activity^2^.

We also noted that just based on the gradient of the neuron transfer-function, trial-by-trial variability could be roughly divided into three regimes (Fig. 3b). Above 5 Hz output rate, the slope of output firing rate vs trial-by-trial variability curve was smallest when the network was operating in a PV-dominated regime (Fig. 3b, red dots) and the slope was largest in an SST-dominated regime (Fig. 3b blue dots). Below 5 Hz firing rate (Fig. 3b, gray dots), we investigated the effect of the trial-by-trial input covariance matrix on the trial-by-trial output variability. We chose a low firing rate region because in this regime all neuron populations were spiking and influenced the output variability. For the high firing rate region, the system degenerated into a lower dimension, i.e. reverting to the E-I network (Fig. 2c–f), when either PV or SST neurons were almost silent (Supplementary Fig. S2a). However, the results of trial-by-trial variability transfer were similar for both regions (Fig. 3 and Supplementary Fig. S2).

We varied the trial-by-trial input balance or trial-by-trial covariance while keeping the mean of inputs constant (Fig. 3c). To make a fair comparison, we chose three different mean input configurations such that the network worked in either PV-dominated (Fig. 3c, top) or SST-dominated (Fig. 3c, middle) or both controlling (Fig. 3c, bottom) regime while having approximately the same output firing rate (E population). Spiking activity and firing rate for the three operating regimes are shown in Supplementary Fig. S1a–c.

To analyze the effect of covariance, we assumed a fixed trial-by-trial balance to the different neuron populations where the input point cloud was in the region of correlated inputs to both E-P, E-S pairs (Fig. 3d, i). Next, we varied the covariance between different pairs of populations (Fig. 3c). In the PV-dominated regime (Fig. 3c top row), increasing ${\sigma }_{{{{{{{{\rm{EP}}}}}}}}}^{{{{{{{{\rm{in}}}}}}}}}$ decreased the trial-by-trial variance, whereas ${\sigma }_{{{{{{{{\rm{ES}}}}}}}}}^{{{{{{{{\rm{in}}}}}}}}}$ had a much smaller effect. In the SST-dominated regime (Fig. 3c middle row) increasing ${\sigma }_{{{{{{{{\rm{ES}}}}}}}}}^{{{{{{{{\rm{in}}}}}}}}}$ decreased the trial-by-trial variance, whereas ${\sigma }_{{{{{{{{\rm{EP}}}}}}}}}^{{{{{{{{\rm{in}}}}}}}}}$ had a much smaller effect. Finally, when both PV and SST neurons affected the network activity, an increase in both ${\sigma }_{{{{{{{{\rm{EP}}}}}}}}}^{{{{{{{{\rm{in}}}}}}}}}$ and ${\sigma }_{{{{{{{{\rm{ES}}}}}}}}}^{{{{{{{{\rm{in}}}}}}}}}$ resulted in a decrease in the output trial-by-trial variability (Fig. 3c bottom row). These results show that the covariance between inputs to the three neuron populations affected the trial-by-trial variability in a state-dependent manner.

The trial-by-trial input balance (orientation of the input point cloud) played a major role in controlling the trial-by-trial output variance of the excitatory population (Fig. 3d). To quantify the effect, we varied the ratio of input variances while keeping the covariance fixed such that the input point cloud ranged from completely aligned to orthogonal to the iso-firing rate manifolds (Fig. 3d) similar to the 2-dimensional case (Fig. 2c, f). In the PV-dominated regime (Fig. 3d top), the orientation for input point cloud with the lowest trial-by-trial output variability was in the region where inputs to E-P pair (${\sigma }_{{{{{{{{\rm{E/P}}}}}}}}}^{{{{{{{{\rm{in}}}}}}}}}$) were positively aligned and to E-S pair (${\sigma }_{{{{{{{{\rm{E/S}}}}}}}}}^{{{{{{{{\rm{in}}}}}}}}}$) negatively aligned. Consequently, anti-correlating the trial-by-trial input rates (negative balance) of the E-P pair induced a large change of trial-by-trial variability (Fig. 3d top, see also Fig. 2f for 2-D case). As expected, the situation reversed for the SST-dominated regime (Fig. 3d middle). Finally, both ${\sigma }_{{{{{{{{\rm{E/P}}}}}}}}}^{{{{{{{{\rm{in}}}}}}}}}$ and ${\sigma }_{{{{{{{{\rm{E/S}}}}}}}}}^{{{{{{{{\rm{in}}}}}}}}}$ contributed to the output variance with comparable strength in the regime where both PV and SST interneurons shaped the network response. In this regime, minimum output variability requires that either E-P balance is negative while E-S balance is positive or vice versa (Fig. 3d bottom).

### Trial-by-trial variability: Network simulation

**Fig. 4:**
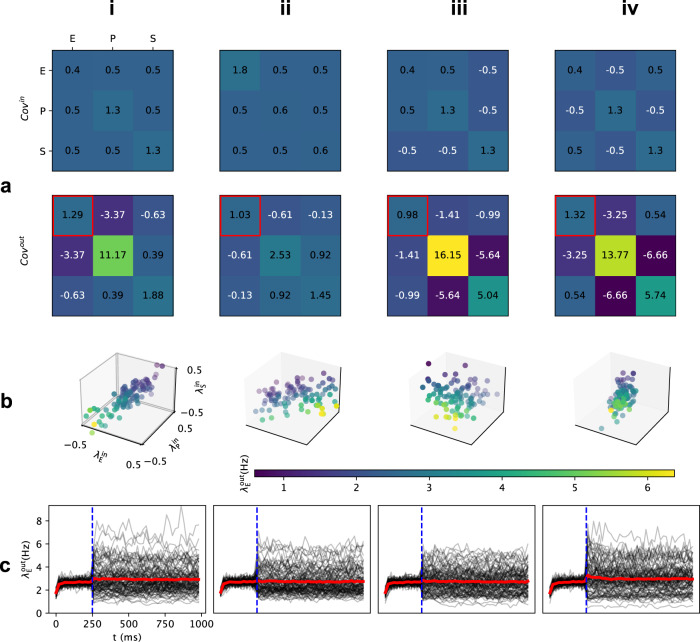
Simulation of trial-by-trial variability controlled by input balance in a PV-dominated regime. **a** top The input covariance matrix for sampling input point clouds (**b**). From **i** to **ii**, the trial-by-trial input balance between E and SST/PV populations were increased. In column **iii** and **iv**, negative covaraince in Cov^in^ denotes the negative trial-by-trial balance as shown in Fig. 2f. The network operated in a state corresponding to the top row in Fig. 3d. **a** bottom Output covariance matrix across 100 trials where inputs for each trial were sampled from the covariance matrix in **a** top. The output variance of the excitatory population is indicated with a red square. (**b**) Trial-by-trial input point clouds sampled from given covariance matrix as columns **i** to **iv** in **a** top. (axes are normalized to the maximal value in Fig. 1d) **c** Peristimulus time histogram (PSTH) of excitatory population response. Black lines: individual trial. Red line: average response over 100 trials. Stimulus (which involved only a change in the variance and/or covariance without any change in the mean) was provided at 250 ms, and the output covariance matrix in the panel **a** bottom was calculated for the last 500 ms.

To confirm the analysis above derived from interpolated neuron transfer-function, we simulated the stimulus-response of the EPSV network (see Methods section: Trial-by-trial variability of the input). We tuned the EPSV network in a PV-dominated regime and stimulated the three neuron populations with inputs sampled from a multivariate normal distribution with a specific covariance matrix (Fig. 4a, top row, and b). In each trial, the network responded with a different output rate (Fig. 4c). To quantify the trial-by-trial variability we measured the covariance matrix from the activity of the three populations (Fig. 4a, bottom row).

First, we simulated the effect of the trial-by-trial balance while keeping other input variables fixed (Fig. 4a, top row, and b). We assumed that the total variance remains constant (i.e. the sum of diagonal in Fig. 4a, top row). The results are consistent with the estimate of trial-by-trial variance derived from the neuron transfer-function. For instance, changing the trial-by-trial balance of inputs to E and P neurons (by an increase of trial-by-trial input variance to the excitatory population while decreasing the variance of input to the PV population) resulted in a decrease in the output variability (Fig. 4 a, i and ii). In this example, a change in the input balance altered the alignment of the input point cloud with the iso-firing rate surfaces, therefore, we observed a decrease in the trial-by-trial output variance.

To further illustrate the effect of the orientation of the input point cloud (or the ratio of input variances), we simulated the network response when the input point cloud had a negative slope (Fig. 4b, iii and iv). In the PV-dominated regime, anti-correlated inputs to E-S pair reduced the output trial-by-trial variability (Fig. 4c, iii). However, when the input cloud slope was negative in the E-P dimensions, trial-by-trial output variability was increased (compare Fig. 4c, ii and iv), consistent with the results obtained using neuron transfer-function (Fig. 3d, top).

**Fig. 5:**
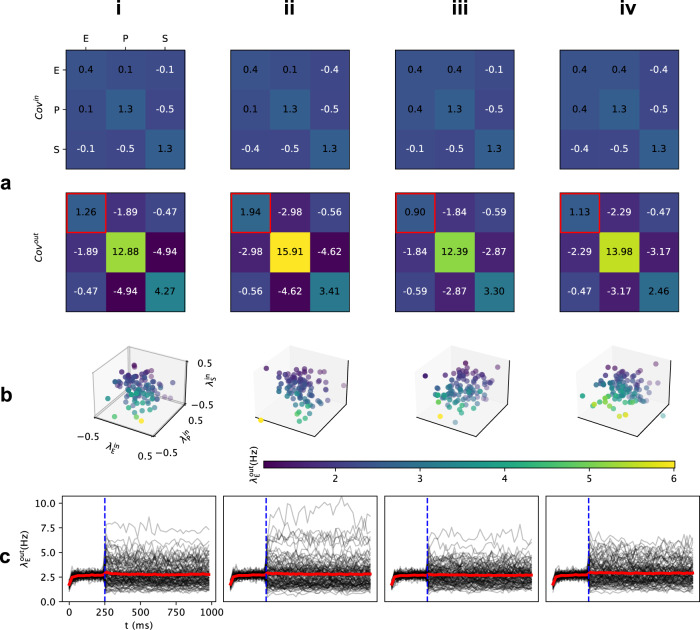
Simulation of trial-by-trial variability controlled by input covariance in a PV-dominated regime. **a** The input covariance matrix for sampling input point clouds (**b**). From **i** to **ii** (**iii**), the trial-by-trial input covariance between E and SST (PV) populations were increased. In column **iv**, covariances between E-P and E-S pairs were both increased. **a** bottom Output covariance matrix across 100 trials where inputs for each trial were sampled from the covariance matrix in **a** top. The output variance of the excitatory population is indicated with a red square. **b** Trial-by-trial input point clouds sampled from given covariance matrix as columns **i** to **iv** in **a** top. (axes are normalized to the maximal value in Fig. 1d) **c** Peristimulus time histogram (PSTH) of excitatory population response. Black lines: individual trial. Red line: average response over 100 trials. Stimulus (which involved only a change in the variance and/or covariance without any change in the mean) was provided at 250 ms, and the output covariance matrix in the **a** bottom was calculated for the last 500 ms.

Next, we varied the trial-by-trial covariance between the inputs to the three neuron populations while fixing all other variables (Fig. 5a, top row). The effect of input covariances is best seen when the input cloud is aligned to iso-firing rate surfaces. Therefore, we tuned the input balances accordingly (see diagonal in Fig. 5a, top row and Fig. 3d, ii). With these variance settings, an increase in the covariance between inputs to E and S populations resulted in a large increase in the output variance of the excitatory population (compare columns I and IV in Fig. 5a, top row). The discrepancy between simulation and results from neuron-transfer interpolated was due to the outlier (yellow dot in Fig. 5b, ii) given limited sampling size. Because we had tuned our network in a PV-dominated regime, an increase in the covariance between inputs to E and P populations resulted in a decrease in the variance of E population (compare Fig. 5c, i and ii). Finally, when we increased both the covariance (${\sigma }_{{{{{{{{\rm{ES}}}}}}}}}^{{{{{{{{\rm{in}}}}}}}}}$ and ${\sigma }_{{{{{{{{\rm{EP}}}}}}}}}^{{{{{{{{\rm{in}}}}}}}}}$), we also observed a decrease in the trial-by-trial variability of E neurons (compare Fig. 5 column i and iv). In general, consistent with our estimates from the neuron transfer-function, in numerical simulations of evoked responses we found that indeed, trial-by-trial variability could be controlled by varying the balance and covariance of trial-by-trial inputs to the different neuron types.

Note that Fig. 5 column iv had a larger output variance compared to Fig. 5 column iii which is not exactly the same with Fig. 3c, middle panel top row. This is due to linear interpolation of neuron transfer-function and limited sampling size for simulations while the actual underlying iso-firing rate surfaces are nonlinear Fig. 1d, right. Simulations of trial-by-trial variability in SST-dominated regime (Supplementary Fig. S4) were also consistent with the estimations made from neuron transfer-function (Fig. 3c, middle row). For the high firing rate region, simulations of trial-by-trial variability in PV-dominated regime (Supplementary Fig. S3) had similar result as in low firing rate region (Fig. 5 and Fig. 4) and these results are consistent with estimations made from neuron transfer-function (Supplementary Fig. S2b, c top row).

### Trial-by-trial variability of inhibitory neurons

In our model, the change of trial-by-trial output variability accompanied different correlations between E, P, and S populations (Fig. 5a, bottom and Fig. 4a, bottom). Increasing the trial-by-trial input variance to the excitatory population did not necessarily cause a corresponding increase in trial-by-trial output variance (compare Fig. 4a, i and ii). Instead, the outputs of different populations were less correlated (compare the output covariance between E and P/S populations in Fig. 4a, i and ii). In the PV-dominated regime, the trial-by-trial output variability (E population) was mainly controlled by the high-firing level PV neurons. Therefore, an increase or decrease in output variance was accompanied by a larger or smaller anti-correlation between the outputs of the E-P pair (compare columns in Fig. 5). The network connectivity generated negative correlations between the outputs of E-I pairs across trials. Such a negative correlation could be enhanced or quenched by the distribution of inputs across trials. Consequently, the interneurons (PV and SST neurons) contributed more or less to output variance (E population).

**Fig. 6:**
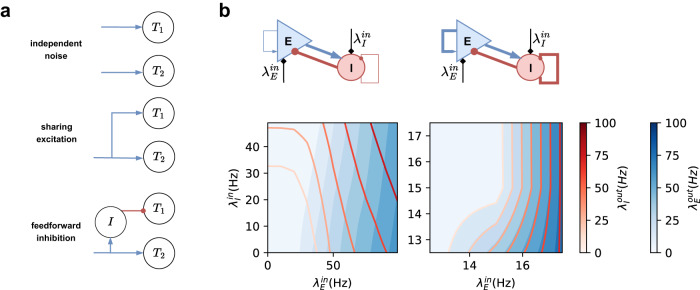
Mechanism of trial-by-trial variability control. **a** Trial-by-trial input distribution modulated by feed-forward input connectivity. (top) Two target populations receive independent background input. (middle) Target populations receive shared excitatory input. (bottom) Target populations receive shared input with opposite signs. **b** Contours of transfer-function (blue for ${\lambda }_{{{{{{{{\rm{E}}}}}}}}}^{{{{{{{{\rm{out}}}}}}}}}$ and red for ${\lambda }_{I}^{{{{{{{{\rm{out}}}}}}}}}$) in a weak recurrently connected E-I network. In this configuration iso-firing rate lines between Exc and Inh populations do not align. **b** In a strong recurrently connected E-I network. In this configuration iso-firing rate lines between Exc and Inh populations align.

Whether the variability of inhibitory interneurons changes in the same way as the variability of the excitatory population depends on the network connectivity. In our model, the output variability of inhibitory populations depends on how their iso-firing rate manifold is aligned with that of the excitatory population. To illustrate this, we considered a two-population E-I network. This network can operate in two regimes—weak self-coupling (or strong mutual interactions) and strong self-coupling (weak mutual interactions). For the weak self-coupling regime, E-I populations had a strong mutual interaction such that the change of one population influenced the other (Fig. 6b, left column). In this regime, E and I iso-firing rate lines were not aligned, therefore a decrease in the variability of the excitatory population will be accompanied by an increase in the variability of the inhibitory population. A similar argument has been made by ref. ^4^. By contrast, in the strong self-coupling regime, the output rate of a population did not depend on the input from the other population (Fig. 6b, right column), and iso-firing rate lines of both E and I populations were aligned. Therefore, a decrease in the variability of the excitatory population will be accompanied by a corresponding decrease in the variability of the inhibitory population.

## Discussion

Here we have investigated how the mean, variance, and covariances of trial-by-trial inputs affect the trial-by-trial variability of the neocortical activity. Transfer of input distribution to output is mediated by the neuron transfer-function. The number of distinct neuron types in a network determines the dimensionality of the neuron transfer-function. For the neocortical microcircuit with one excitatory and two or three inhibitory populations, the neuron transfer-function becomes 3- or 4-dimensional. As the dimensionality of the neuron transfer-function increases beyond one, we need to consider not only the variance of the input but also the ratio of input variances and covariance between all pairs of neuron types. Here, we have unraveled how the ratio of variance and covariance of inputs to excitatory, PV, and SST neurons affect the trial-by-trial variance of cortical activity.

For the neocortex network model, the non-linearity of steady state neuron transfer-function resulted in non-trivial relation between output rate and output variance (Fig. 3a). For unstructured inputs (no correlation between inputs to different populations) output variance was higher in the SST-dominated regime compared to the PV-dominated regime. Next, output variance could be significantly altered without any change in the output rate by tuning the input covariance matrix (Fig. 3c). For the PV-dominated regime, output variance decreased with across-trial correlated inputs to E-P pair and increased when anti-correlated. The results were similar for E-S pair in the SST-dominated regime. That is, SST and PV neurons contribute in the same way. However, when both interneurons are controlling the dynamics minimum output variability requires that E-S and E-P balances have opposite sign. In general, the shape and orientation of input distribution across trials should align with the iso-firing rate manifold for the corresponding network state to reach low output variability. Finally, the non-linearity of the neuron transfer-function implies that the input covariance matrix may never fully align with the iso-firing rate manifolds hence the trial-by-trial output variability is inevitable. The orientation of the input cloud, i.e. trial-by-trial input balance, is the main predictor of trial-by-trial variability as it aligned the input point cloud to the iso-firing rate manifold. Once such an alignment is achieved, covariance can be varied to further modulate the trial-by-trial variability.

### Control of trial-by-trial variability

Trial-by-trial variability is necessary for behavior^6^ therefore it is crucial that it can be varied in a context-, behavioral state-, and task-dependent manner. For instance, during the early stages of learning, we would like higher variability to explore the state space but once the task is learned animals should reduce variability in their behavior.

Our model suggests that feedforward connectivity provides a natural way to alter the input statistics. If the two target populations receive independent inputs (Fig. 6a, top), there is no correlation between inputs, and the output variability is solely controlled by the trial-by-trial input variance. In such a scenario, feedforward weights can modulate the input variance: the larger the feedforward weights, the larger the trial-by-trial input variance, and vice versa. When the target populations, e.g. E and P populations, share a certain level of input excitation (Fig. 6a, middle) due to feedforward divergent connections from the same sources (thalamus), their inputs co-vary and the slope of the input point cloud is positive. In this scenario, the degree of shared input controls the degree of covariance (Fig. 3c and Fig. 5), and feedforward input strength controls the input balance i.e. the slope of the input point cloud (Fig. 3d, and Fig. 4 column i, ii). The slope of the input cloud could be inverted if the common input to one of the two populations is mediated via an inhibitory interneuron (Fig. 6a, bottom). Thus, the structure and strengths of feedforward connectivity provide a natural control over the distribution of received inputs across trials. Feedforward input synapses can be learned through plasticity mechanisms so that the animal may reach the desired degree of trial-by-trial variability in their activity and thereby in their behavior. Furthermore, modulation of neuronal excitability and synaptic strength by neuromodulators can provide a context-dependent control of the trial-by-trial variability.

In parallel to the feedforward input structure, changes in the local activity dynamics can also modulate trial-by-trial variability by changing the gradient of the neuron transfer-function and the curvature of iso-firing rate curves. At the simplest, this can be achieved by switching the network between SST-dominated and PV-dominated states. Furthermore, intrinsic excitability and synaptic plasticity, when changing the network between weak (Fig. 6b, left) and strong connection (Fig. 6b, right) regimes, can modulate trial-by-trial variability by altering the whole landscape of neuron transfer-function.

### Relationship with other explanations of trial-by-trial variability control

Previous models of trial-by-trial variability have mostly focused on the dynamics of recurrent connectivity^14–16, 30^. However, trial-by-trial variability is also shaped by external inputs^3,31^ and attention signals^4,16^. More specifically, within-trial correlations in the feedforward inputs can modulate the trial-by-trial variability^18^.

Here we have further explored the role of feedforward inputs in shaping the trial-by-trial variability in a network with three different interneuron populations. While focusing on the feedforward input rate covariance and variances, we have not ignored the role of recurrent activity dynamics. The neuron transfer-function responsible for the transfer of input variance and covariances is shaped by the recurrent activity. That is, the neuron transfer-function we have used is not the same as we may estimate by current injections in a silent network (e.g. in vitro slices). When the network is operating in different regimes such as an inhibition stabilized network (ISN), the results may change but only to the extent that in an ISN regime neuron transfer-function may be quite different^32^. Thus, the approach of using the neuron transfer-functions allows us to combine both input statistics and recurrent activity state.

Thus, in contrast to previous work where the focus was on the input correlation at the spiking activity level, we showed that interneurons contribute to the rate variability in three ways by modulating: (1) the effective transfer-function of the neuron, (2) the operating regime of the network and (3) the trial-by-trial input variance and covariances.

### Function of interneurons

Given the diversity of interneurons in the brain, there is an impetus to identify specific functions of each interneuron subtype^19^. Classically, the function of interneurons is thought in terms of control of gain modulation^33,34^, control of network activity state^35^, decorrelation of network activity^26,36^, gating of input^34,37^ and control of dynamics of oscillations^21,23^. Moreover, different interneurons such as PV, SST, and VIP have been implicated in specific functions such as layer-specific control of excitation-inhibition balance^38^ and synchronization of gamma-band oscillations^39^. Here we have identified a new role of interneurons—in controlling the trial-by-trial variability. In particular, for the first time, we highlight the importance of the structure of task-related feedforward inputs to the interneurons. Moreover, multiple interneuron types provide more means to control the trial-by-trial variability. Because the cortical networks can switch between SST and PV-dominated regimes depending on the context^40^ and attention levels^41,42^ of the animal, interneurons provide mechanisms to modulate trial-by-trial variability in a context-, task-, and behavioral state-dependent manner.

### Model limitations

In the model, we have a number of assumptions e.g. all neurons were modeled as point neurons and connectivity was independent of spatial distance among neurons. However, the most crucial assumption we made is that the network is operating in a near asynchronous-irregular state. In such a state, it is straightforward to relate the input variance and covariance to output variance. But when the network is operating in an oscillatory state (Supplementary S1d), our single neuron transfer-function approach may not be sufficient. Given the oscillations, we will also have to consider the serial correlations in the input spiking activity and input oscillations. Moreover, inputs with serial correlations or oscillations could affect the downstream network by entrainment or resonance^43^. While interesting, this is beyond the scope of the current manuscript.

Here we followed an approach similar to the use of linear-response theory to estimate the transfer of input correlations^25,27^. Neuron transfer-function is informative about the output trial-by-trial variability when the input is small enough and both magnitude and history of the input do not affect the neuron transfer-function. If these conditions are not met, we will have to resort to full network simulations. Furthermore, when variability of the ongoing activity is high, neuron transfer-function may not give an accurate estimate of the trial-by-trial variability. Indeed, our neuron transfer-function approach ignores variability arising from the fluctuations in the background activity of the network.

We used the data from ref. ^29^ to tune the baseline firing rate of our network. It can be argued that in that study there was a bias towards high firing rate neurons. If we were to assume a lower baseline firing rate, for all the different neuron subtypes, to estimate the transfer-function we will have to reduce the range of negative modulation and increase the range of positive modulation. This might affect the results in a quantitative manner. Unless at very low-firing rates the network shows some kind of hysteresis-like behavior we do not expect our results to change in a qualitative manner.

Next, our conclusion that input covariance and balance are crucial for the control of trial-by-trial variability is contingent on the fact that the iso-firing rate surfaces are continuous. It is possible that due to non-linear interactions, iso-firing rate regions in the input space appear as small islands. In such a scenario, the effect of input variance and covariance also become contingent on the operating point or the mean output firing rate.

We have shown that increasing the covariance of inputs to E and PV or SST neurons reduces the trial-by-trial variability. This is true when input variance is small enough such that the input cloud can align with the iso-firing rate manifold. When input variance becomes larger, or the curvature of iso-firing rate surfaces is high, an increase in the covariance can misalign the input point cloud from the iso-firing rate cloud and result in a higher trial-by-trial variability. Moreover, when ongoing activity of the network is high, it might end up altering the input statistics rendering our predictions wrong.

Here we have ignored the fact that there may also be within-trial variability. Within-trial variability is largely determined by the statistics of the ongoing activity in the network. Given that network may exhibit different activity states, i.e. PV- or SST- dominated regimes, these interneurons can affect the within-trial variability. However, it is important to determine whether the interaction between ongoing activity and stimulus is additive or multiplicative. In the later cases, within-trial variability may influence trial-by-trial variability. There is some suggestion that PV and SST neurons have multiplicative and additive effects on the stimulus responses^44^. Therefore, we expect that PV and SST neurons will also affect within-trial activity but addressing this question is beyond the scope of this manuscript.

#### Model predictions and model verification

Like any good computational model, we have also made several simplifications in our model (see Methods). However, our model still captures a number of crucial biological details and makes testable predictions. First and foremost, our results suggest that to better understand the modulation of trial-by-trial variability we should measure the variability and co-variability of inputs to different neuron populations. A straightforward prediction of our model is that when inputs to excitatory and inhibitory neurons are correlated, an increase in the input variance may not increase the output trial-by-trial variability.

While simultaneous measurements of excitatory and inhibitory inputs to a neuron is difficult, indirect (and non-simultaneous) measurements from cortical neurons suggest a high correlation between excitatory and inhibitory inputs^45,46^. Such a correlation between excitatory and inhibitory inputs should lead to lower trial-by-trial variability compared to uncorrelated cases. That is, our model predicts high correlation between excitatory and inhibitory activity when variability is reduced. The feedforward inhibition circuit motif ensures that excitatory and inhibitory inputs to a neuron are correlated. Therefore, we predict that blocking of feedforward inhibitory circuit should not only alter the response mean firing rate but also increase the trial-by-trial variability.

Recently, it has become possible to elicit behavior by optical stimulation of selected neurons which were also activated by an external stimulus^47^. Our model predicts that under optogenetic activation/inactivation the trial-by-trial output variability should be different from that observed during sensory stimulation conditions because input structures are qualitatively different in the two experiments. With the current optogenetic stimulation methods, it is also feasible (though tedious) to mimic our simulation approach in vivo and verify our model results.

Our model also predicts that there will be higher trial-by-trial variability when the network is operating in an SST-dominated state compared to PV-dominated state. Interestingly, a reduction in SST activity is often seen in experiments, e.g. from non-whisking to whisking in the barrel cortex of mice^29^.

Neuromodulators can alter synaptic strengths and neuronal excitability. In our model, a change in the feedforward synaptic weights implies a change in the input variance and a change in neuronal excitability implies rotation or shift of the neuron iso-firing rate manifolds. Therefore, the effect of any neuromodulator on trial-by-trial variability should be non-monotonic because only for a specific amount of neuromodulator it may be possible to align the input cloud with the iso-firing rate manifold.

## Methods

### Neuron model

The neurons were realized as the adaptive exponential integrate and firing model^48^:

2 $${C}_{{{{{{{{\rm{m}}}}}}}}}\frac{dV}{dt} = 	-{g}_{{{{{{{{\rm{L}}}}}}}}}(V-{{{{{{{{\rm{E}}}}}}}}}_{{{{{{{{\rm{L}}}}}}}}})+{g}_{{{{{{{{\rm{L}}}}}}}}}{{{\Delta }}}_{{{{{{{{\rm{T}}}}}}}}}exp\left[\frac{V-{{{{{{{{\rm{V}}}}}}}}}_{{{{{{{{\rm{th}}}}}}}}}}{{{{\Delta }}}_{{{{{{{{\rm{T}}}}}}}}}}\right]\\ 	-{g}_{{{{{{{{\rm{e}}}}}}}}}(t)(V-{{{{{{{{\rm{E}}}}}}}}}_{{{{{{{{\rm{e}}}}}}}}})-{g}_{{{{{{{{\rm{i}}}}}}}}}(t)(V-{{{{{{{{\rm{E}}}}}}}}}_{{{{{{{{\rm{i}}}}}}}}})-w+{I}_{{{{{{{{\rm{e}}}}}}}}}\\ {\tau }_{w}\frac{dw}{dt} = 	\,a(V-{{{{{{{{\rm{E}}}}}}}}}_{{{{{{{{\rm{L}}}}}}}}})-w$$

The equation denotes the subthreshold dynamics of membrane potential *V* and spike-adaptation current *w*. When the membrane potential reached the threshold *V* ≥ V_th_, the neuron elicited a spike and its membrane potential was reset *V* = V_r_ and an adaptation current was added *w*_*t*+_ = *w*_*t*−_ + b. In addition to external current injection *I*_e_, the neuron was driven by conductance based excitatory and inhibitory synaptic inputs (see synapse model below).

There are three categories of parameters: membrane parameters include capacity C_m_, leak reversal potential E_L_, leak conductance *g*_L_, and constant external input current *I*_e_ = 0; spike parameters include refractory period *t*_r_, subthreshold adaptation a, spike-triggered adaptation b, slope factor Δ_T_, spike initiation threshold V_th_, and adaptation time constant *τ*_*w*_; synaptic parameters include excitatory reversal potential E_e_, inhibitory reversal potential E_i_, rise time of excitatory synaptic alpha function *τ*_e_ and rise time of inhibitory synaptic alpha function *τ*_i_.

Neuron and synapse parameters are provided in the Table 1^21^.

**Table 1:** Neuron parameters^*^.

Type	V_r_ mV	E_L_ mV	*g*_L_ nS	V_th_ mV	a nS	b pA	Δ_T_ ms	*τ*_*w*_ ms	*τ*_e_ ms	*τ*_i_ ms
PC	−66.4	−70.0	20.3	−41.5	2.0	4.0	2.0	120.0	0.22	5.0
PV	−67.4	−70.0	77.1	−41.6	–	–	–	–	0.2	5.5
SST	−59.9	−70.0	21.4	−41.8	2.0	4.0	2.0	120.0	0.29	9.1
VIP	−65.7	−70.0	26.6	−43.7	−1.0	19.0	2.0	120.0	0.28	12.2

^*^C_m_ = 2*e*3 mV, E_e_ = 0 mV, E_i_ = − 85 mV, *t*_r_ = 2.0 ms for all

### Synapse model

Neurons were connected using conductance-based synapses. Each incoming spike resulted in a conductance transient *g*(*t*) (excitatory *g*_e_(*t*)) or inhibitory *g*_i_(*t*) which decayed exponentially with a time constant *τ*_syn_ (excitatory *τ*_e_ or inhibitory *τ*_i_):

3 $$g(t)=\mathop{\sum}\limits_{i}\overline{{{{{{{{{\rm{g}}}}}}}}}_{{{{{{{{\rm{syn}}}}}}}}}}exp\left(-\frac{t-{t}_{i}}{{\tau }_{{{{{{{{\rm{syn}}}}}}}}}}\right)H(t-{t}_{i})$$

where *t*_*i*_ is the arrival time of *i*^*th*^ spike and *H* is the heaviside step function.

### Network model

**Fig. 7:**
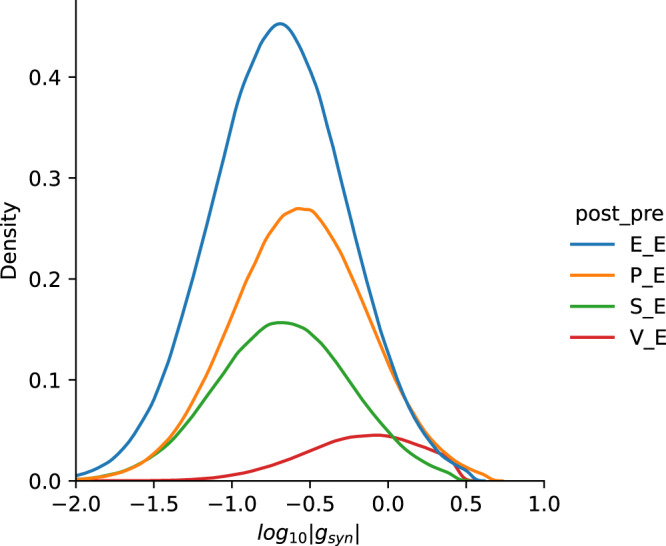
Distribution of non-zero synaptic conductance. Synaptic strength (peak conductance for different types of synapses were drawn from a specific log-normal distribution for each type of connection shown in the figure).

The network consists of 4800 neurons with 3600 excitatory, 480 PV, 360 SST, and 360 VIP neurons^49^. Neuronal connectivity parameters (see Table 2) were taken from ref. ^22^ with modifications. Given the size of the network, neurons were connected in a distance-independent manner that each pair of neurons has probability *p*_*ts*_ to form a connection depending on the types of source and target as in Table 2 left. Each connection was generated independently and autapses were not allowed. For synaptic conductance, we first chose the value $\overline{{{{{{{{{\rm{g}}}}}}}}}_{{{{{{{{\rm{syn}}}}}}}}}}$ for each pair of connections as in Table 2 right. Next, we scaled each synaptic weight by a number randomly drawn from a log-normal distribution (*μ* = 0, *σ* = 1) and upper bounded the weights by 0.5 mV (−2.0 mV) for EPSP (IPSP) at *V* =−55 mV. The distribution of excitatory conductance is shown in Fig. 7. In the main text, all results are shown for a network with log-normal synaptic weight distribution. All synapses had a conduction delay of 2ms.

**Table 2:** Network connectivity parameters.

	Src							
		Conn.	Prob.			Cond.	(nS)	
Tar	PC	PV	SST	VIP	PC	PV	SST	VIP
PC	0.1	0.6	0.55	–	0.20	0.08	0.16	–
PV	0.45	0.5	0.6	–	0.27	0.46	0.45	–
SST	0.35	–	0.5	0.5	0.21	–	–	0.07
VIP	0.1	–	0.45	0.6	0.78	–	0.07	–

### Baseline input

To mimic the ongoing activity, each neuron received excitatory inputs as uncorrelated homogeneous Poisson spike trains from 20 presynaptic neurons. These inputs represent long-range excitatory projections to the network. The weight of external excitatory synapses was 50nS. The input rates to different neuron-type populations, $({\lambda }_{{{{{{{{\rm{E}}}}}}}}}^{{{{{{{{\rm{base}}}}}}}}},{\lambda }_{{{{{{{{\rm{P}}}}}}}}}^{{{{{{{{\rm{base}}}}}}}}},{\lambda }_{{{{{{{{\rm{S}}}}}}}}}^{{{{{{{{\rm{base}}}}}}}}},{\lambda }_{{{{{{{{\rm{V}}}}}}}}}^{{{{{{{{\rm{base}}}}}}}}})=(16.0,90.0,9.3,12.0)$ Hz, were tuned to obtain a baseline firing rate around 2.5 Hz for excitatory and SST neurons, and around 14 Hz for PV and VIP neurons (spontaneous activity level of free whiskering mice^29^). Note the baseline network state was PV-dominated in our model (Supplementary S1 top row).

### Stimulus evoked input

To mimic stimulus-evoked inputs, each population reveived additional inputs with rates ${{\Delta }}{\lambda }_{{{{{{{{\rm{E}}}}}}}}}^{{{{{{{{\rm{in}}}}}}}}},{{\Delta }}{\lambda }_{{{{{{{{\rm{P}}}}}}}}}^{{{{{{{{\rm{in}}}}}}}}},{{\Delta }}{\lambda }_{{{{{{{{\rm{S}}}}}}}}}^{{{{{{{{\rm{in}}}}}}}}},{{\Delta }}{\lambda }_{{{{{{{{\rm{V}}}}}}}}}^{{{{{{{{\rm{in}}}}}}}}}$ on top of the baseline inputs as shown in Fig. 1. The final inputs, baseline plus addtional inputs, were excitatory uncorrelated homogeneous Poisson spike trains with rates ${\lambda }_{{{{{{{{\rm{i}}}}}}}}}^{{{{{{{{\rm{in}}}}}}}}}={\lambda }_{{{{{{{{\rm{i}}}}}}}}}^{{{{{{{{\rm{base}}}}}}}}}+{{\Delta }}{\lambda }_{{{{{{{{\rm{i}}}}}}}}}^{{{{{{{{\rm{in}}}}}}}}}$. Note the stimulus-evoked inputs could be negative as long as the final input rate was positive. Since SST and VIP neurons are mutually coupled, we assumed that modulatory input to VIP neurons is just inverted input to SST neurons as in^22^ hence reduced the input dimension to three with ${{\Delta }}{\lambda }_{{{{{{{{\rm{V}}}}}}}}}^{{{{{{{{\rm{in}}}}}}}}}=0$. We measured the steady-state response of four populations, ${\lambda }_{{{{{{{{\rm{E}}}}}}}}}^{{{{{{{{\rm{out}}}}}}}}},{\lambda }_{{{{{{{{\rm{P}}}}}}}}}^{{{{{{{{\rm{out}}}}}}}}},{\lambda }_{{{{{{{{\rm{S}}}}}}}}}^{{{{{{{{\rm{out}}}}}}}}},{\lambda }_{{{{{{{{\rm{V}}}}}}}}}^{{{{{{{{\rm{out}}}}}}}}}$, to different levels of modulatory inputs covering a cubic input space. The corresponding output space was restricted due to the interaction between excitation and inhibition as shown in Fig. 1. Because the firing rate of the excitatory population was taken as the output, the transfer-function was formulated as

4 $${\lambda }_{{{{{{{{\rm{E}}}}}}}}}^{{{{{{{{\rm{out}}}}}}}}}={f}_{{{\Delta }}{\lambda }_{{{{{{{{\rm{V}}}}}}}}}^{{{{{{{{\rm{in}}}}}}}}} = 0}({{\Delta }}{\lambda }_{{{{{{{{\rm{E}}}}}}}}}^{{{{{{{{\rm{in}}}}}}}}},{{\Delta }}{\lambda }_{{{{{{{{\rm{P}}}}}}}}}^{{{{{{{{\rm{in}}}}}}}}},{{\Delta }}{\lambda }_{{{{{{{{\rm{S}}}}}}}}}^{{{{{{{{\rm{in}}}}}}}}})$$

(Fig. 1). To obtain a transfer-function with higher resolution, we interpolated the simulated transfer-function with (tri)linear function^50^ implemented in SciPy library^51^. The analysis of variability transformation was derived from this interpolated neuron transfer-function.

### Trial-by-trial variability of the input

Trial-by-trial variability of the modulatory inputs was modeled as a three-dimensional normal distribution (Fig. 3) characterized as:

5 $$\vec{{\mu }^{{{{{{{{\rm{in}}}}}}}}}}=\left[\begin{array}{c}\overline{{\lambda }_{{{{{{{{\rm{E}}}}}}}}}^{{{{{{{{\rm{in}}}}}}}}}}\\ \overline{{\lambda }_{{{{{{{{\rm{P}}}}}}}}}^{{{{{{{{\rm{in}}}}}}}}}}\\ \overline{{\lambda }_{{{{{{{{\rm{S}}}}}}}}}^{{{{{{{{\rm{in}}}}}}}}}}\end{array}\right],{\sigma }^{{{{{{{{\rm{in}}}}}}}}}=\left[\begin{array}{ccc}{\sigma }_{{{{{{{{\rm{EE}}}}}}}}}^{{{{{{{{\rm{in}}}}}}}}}&{\sigma }_{{{{{{{{\rm{EP}}}}}}}}}^{{{{{{{{\rm{in}}}}}}}}}&{\sigma }_{{{{{{{{\rm{ES}}}}}}}}}^{{{{{{{{\rm{in}}}}}}}}}\\ {\sigma }_{{{{{{{{\rm{PE}}}}}}}}}^{{{{{{{{\rm{in}}}}}}}}}&{\sigma }_{{{{{{{{\rm{PP}}}}}}}}}^{{{{{{{{\rm{in}}}}}}}}}&{\sigma }_{{{{{{{{\rm{PS}}}}}}}}}^{{{{{{{{\rm{in}}}}}}}}}\\ {\sigma }_{{{{{{{{\rm{SE}}}}}}}}}^{{{{{{{{\rm{in}}}}}}}}}&{\sigma }_{{{{{{{{\rm{SP}}}}}}}}}^{{{{{{{{\rm{in}}}}}}}}}&{\sigma }_{{{{{{{{\rm{SS}}}}}}}}}^{{{{{{{{\rm{in}}}}}}}}}\end{array}\right]$$

For each setting of mean and covariance matrix, the input rates ${\lambda }_{{{{{{{{\rm{E}}}}}}}}}^{{{{{{{{\rm{in}}}}}}}}},{\lambda }_{{{{{{{{\rm{P}}}}}}}}}^{{{{{{{{\rm{in}}}}}}}}},{\lambda }_{{{{{{{{\rm{S}}}}}}}}}^{{{{{{{{\rm{in}}}}}}}}},{\lambda }_{{{{{{{{\rm{V}}}}}}}}}^{{{{{{{{\rm{in}}}}}}}}}$ were sampled (10000-point input cloud) from a normal distribution with given $\vec{{\mu }^{{{{{{{{\rm{in}}}}}}}}}}$ and *σ*^in^. The corresponding outputs were extrapolated from the neuron transfer-function.

We investigated the transformation of input distribution to output distribution by systematically changing the mean of input rates $\vec{{\mu }^{{{{{{{{\rm{in}}}}}}}}}}$,across-trial balance of input rates ${\sigma }_{{{{{{{{\rm{E/S}}}}}}}}}^{{{{{{{{\rm{in}}}}}}}}},{\sigma }_{{{{{{{{\rm{E/P}}}}}}}}}^{{{{{{{{\rm{in}}}}}}}}},{\sigma }_{{{{{{{{\rm{P/S}}}}}}}}}^{{{{{{{{\rm{in}}}}}}}}}$, and across-trial covariances of input rates ${\sigma }_{{{{{{{{\rm{EP}}}}}}}}}^{{{{{{{{\rm{in}}}}}}}}},{\sigma }_{{{{{{{{\rm{ES}}}}}}}}}^{{{{{{{{\rm{in}}}}}}}}},{\sigma }_{{{{{{{{\rm{PS}}}}}}}}}^{{{{{{{{\rm{in}}}}}}}}}$ (assuming ${\sigma }_{{{{{{{{\rm{EE}}}}}}}}}^{{{{{{{{\rm{in}}}}}}}}}+{\sigma }_{{{{{{{{\rm{PP}}}}}}}}}^{{{{{{{{\rm{in}}}}}}}}}+{\sigma }_{{{{{{{{\rm{SS}}}}}}}}}^{{{{{{{{\rm{in}}}}}}}}}={{{{{{{\rm{const}}}}}}}}$). Anti-correlated trial-by-trial inputs with negative trial-by-trial balance were generated by using negative covariance as in Fig. 4a.

Covariance matrices being not positive semidefinite were discarded. These are marked as white space in the Fig. 3c.

To confirm the analysis, we simulated the three network states in Fig. 3a, right with different covariance matrices. The covariance matrices were chosen to show the trend of variability modulation regarding trial-by-trial covariance and trial-by-trial balance (ratio of input variances): for the former factor, we chose four settings where both pairs, E-P and E-S, had low trial-by-trial input covariance, E-P had large covariance, E-S had large covariance, and both had large covariance (Fig. 5a, upper); for the latter factor, we chose four settings where both pairs had low trial-by-trial input balance, both had high ratio, E-S had negative ratio, and E-P had negative ratio (Fig. 4a, upper).

For each network state (mean input) and covariance matrix, we simulated 100 trials for 1000 ms each with 250 ms preparation to reach the operating point, 250 ms to reach the stable state after injecting modulatory inputs, and last 500 ms were taken as the steady-state response (Fig. 5c and Fig. 4c). Firing rate traces of four populations and corresponding spike raster are illustrated in Supplementary S1. The result of trial-by-trial variability control for the PV-dominated regime is shown in Fig. 5 and Fig. 4, and the result for the SST-dominated regime is given in Supplementary S4.

### Simulation and data analysis tools

The simulations were performed using the NEST simulator^52^. Differential equations were integrated using a fixed timestep of 0.1 ms. The analysis of simulated network activity was done using customized code written in Python. The results were visualized using matplotlib and figurefirst.

#### Statistics and reproducibility

For each simulation, we used the mean firing rate of each population (E, P, and S) in the last 500 ms as the steady-state response rate. Across 100 trials, we calculated the covariance matrix of their steady-state response rate (Fig. 5a, bottom and Fig. 4a, bottom). Diagonal values in the output covariance matrix denote the trial-by-trial variance of each population and the rest values denote trial-by-trial covariance between populations. The results can be replicated using the source code of the model [see link below].

### Reporting summary

Further information on research design is available in the Nature Portfolio Reporting Summary linked to this article.

### Supplementary information

Supplementary Material

Reporting Summary

## Data Availability

Source data for all plots and graphs can be generated from the source code provided in https://github.com/michaelglh/MotifNet.git.

## Code availability

The code for simulating the network and visual illustrations is shared on github https://github.com/michaelglh/MotifNet.git.
