# Supplementary material for: Role of interneuron subtypes in controlling trial-by-trial output variability in the neocortex
Source: Commun Biol. 2023 Aug 25;6:874. doi: 10.1038/s42003-023-05231-0 (PMC10449833; doi:10.1038/s42003-023-05231-0)
Supplement: Supplementary file 1 — Supplementary Material [file 42003_2023_5231_MOESM1_ESM.pdf]

## Supplementary Material

Figure S1: **Firing rate and spike raster for a PV-dominated, SST-dominated, and both influencing network states.**

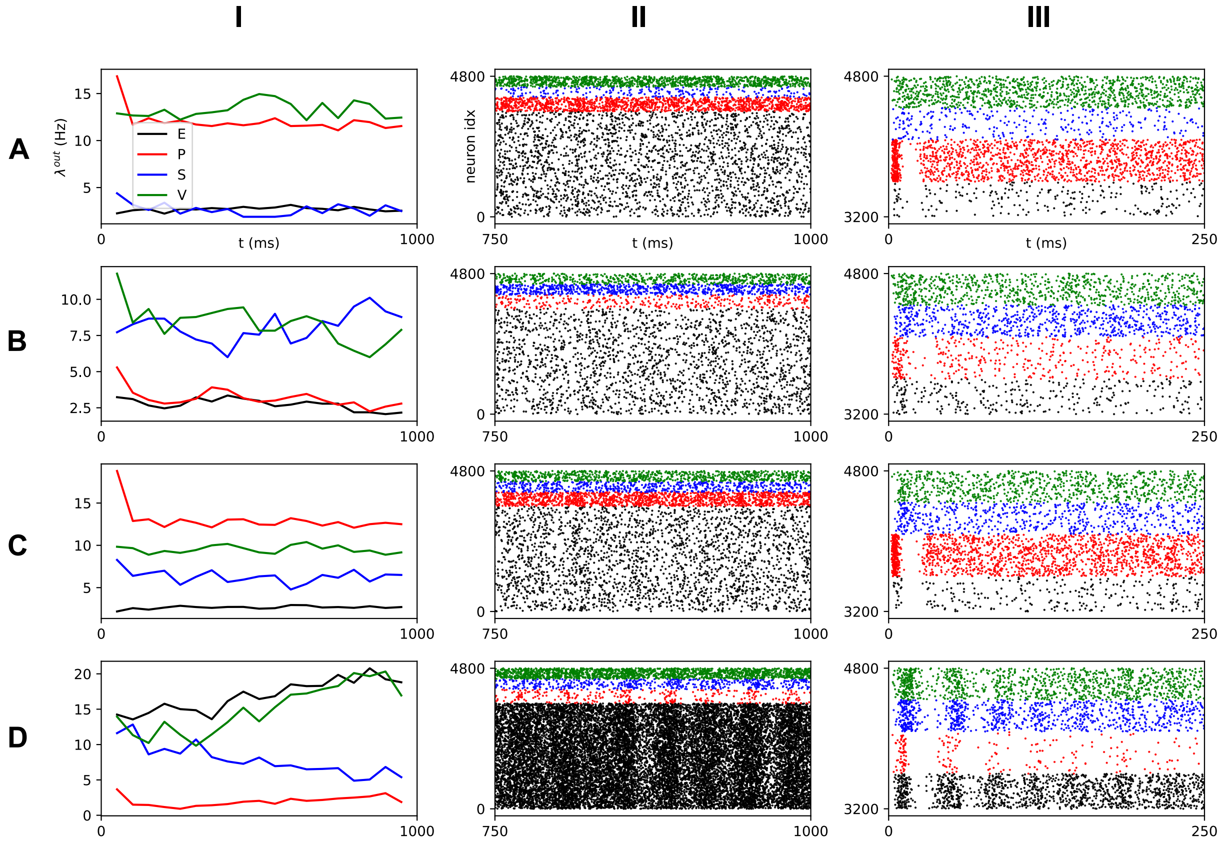


*(**A**-**I**) Average firing rate of four populations in PV-dominated regime (Figure 3C top row) initialized at random state; (**A**-**II**) Spike raster of all 4800 neurons for last 250ms (colors denote the corresponding population in column **I**); (**A**-**III**) Zoom-in of initial 250ms for a subset of the whole network. (**B**,**C**) Same arrangement as in the panel **A** for an SST-dominated and a PV-SST driven regime. (**D**) The network can exhibit stochastic oscillation between populations given strong inputs. In our analysis, we avoided such strong inputs and assumed that the network remained in an asynchronous-irregular activity state..*

Figure S2: **Trial-by-trial variability in EPSV network for high output firing rate (10 Hz) state.**

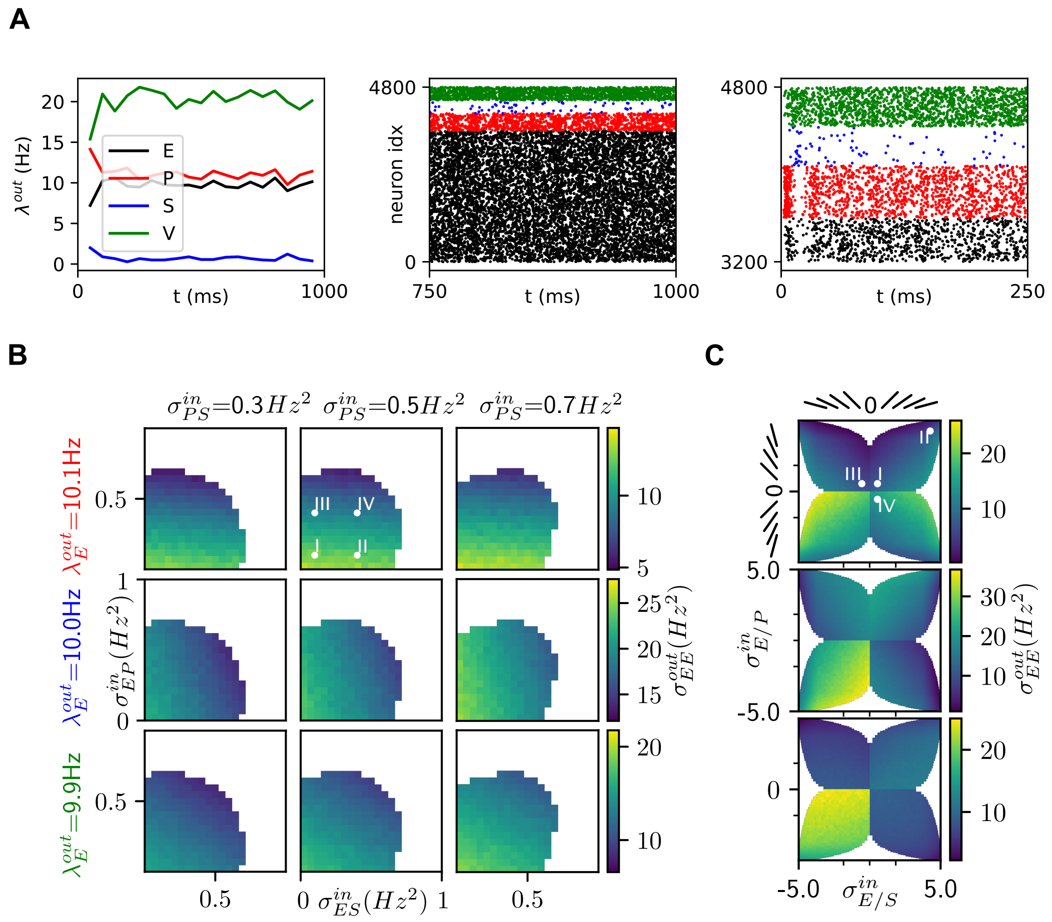


*(**A** left) Average firing rate, of four populations for 1000ms trial in PV-dominated regime. (**A** middle) Spike raster plot of all 4800 neurons for the last 250ms (colors denote the corresponding population in the left panel); (**A** right) Zoom-in of initial 250ms for a subset of the whole network. (**B**) Trial-by-trial output variability as a function of the trial-by-trial input covariance with fixed variance $\sigma^{in}_{EE} = 1.8$, $\sigma^{in}_{PP} = \sigma^{in}_{SS} = 0.6$ in different network regimes (Top: PV-dominated, Middle: SST-dominated, Bottom: both PV-SST driven). (**C**) Trial-by-trial output variability as a function of the trial-by-trial balance with fixed covariance $\sigma^{in}_{PS} = \sigma^{in}_{ES} = \sigma^{in}_{EP} = 0.5$. Slanted bars indicate the orientation of the input point cloud. Roman numerals in the panels **B** and **C** refer to the four cases simulated in Figure S3..*

Figure S3: **Trial-by-trial variability in a PV-dominated regime for high output firing (10 Hz) state.**

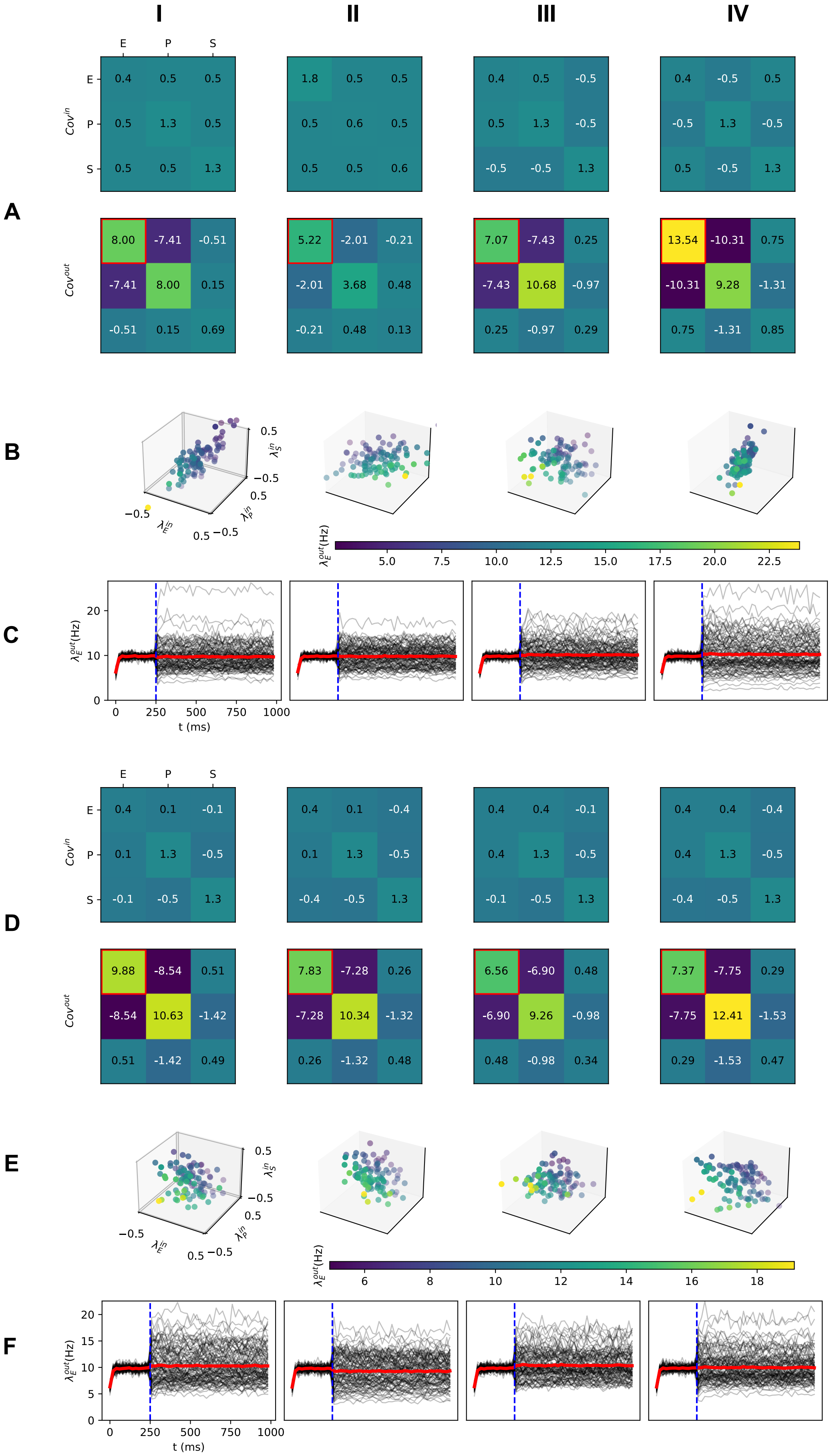

*(**A top**) The input covariance matrix is used to sample inputs across trials **B**. From **I** to **II**, the trial-by-trial input balance between E and SST/PV populations were increased. In column **III** and **IV**, negative variance in $Cov^{in}$ denotes the negative slope of input point clouds as shown in Figure 2F. The network operated in a state corresponding to the middle row in Figure 3D. (**A bottom**) Output covariance matrix across 100 trials where the input for each trial was sampled from the covariance matrix in **A top**. The output variance of the excitatory population is marked with a red square. (**B**) Trial-by-trial input point clouds sampled from given covariance matrix as columns **I** to **IV** in **A top** (axes are normalized to the maximal value in Figure 1D.) **C** PSTH of excitatory population response. Black lines: individual trial. Red line: average response over 100 trials. The stimulus was provided at 250ms, and the output covariance matrix in **A bottom** is calculated for the last 500ms. Note that the stimulus did not involve any change in the input mean, only the input covariance matrix was altered.(**D top**) The input covariance matrix for sampling inputs across different trials. From **I** to **II** (**III**), the trial-by-trial input covariance between E and SST (PV) populations were increased. In column **IV**, both covariances were increased. (**D bottom**) Output covariance matrix across 100 trials where the input for each trial was sampled from the covariance matrix in **D top**. The output variance of the excitatory population is marked with a red square. (**E**) trial-by-trial input point clouds sampled from given covariance matrix as columns **I** to **IV** in **D top** (normalized range). (**F**) PSTH of excitatory population response. Black lines: individual trial. Red line: average response over 100 trials. The stimulus was given at 250ms, and the output covariance matrix in **D bottom** is calculated for the last 500ms.*

*The trend is consistent with Figure 3C middle row. Exact values are slightly different due to linear interpolation of neuron transfer-function and limited sampling size for simulations while the actual underlying iso-firing rate surfaces are nonlinear Figure 1D right..*

Figure S4: **Simulation result of trial-by-trial variability in a SST-dominated regime for low output firing state.**

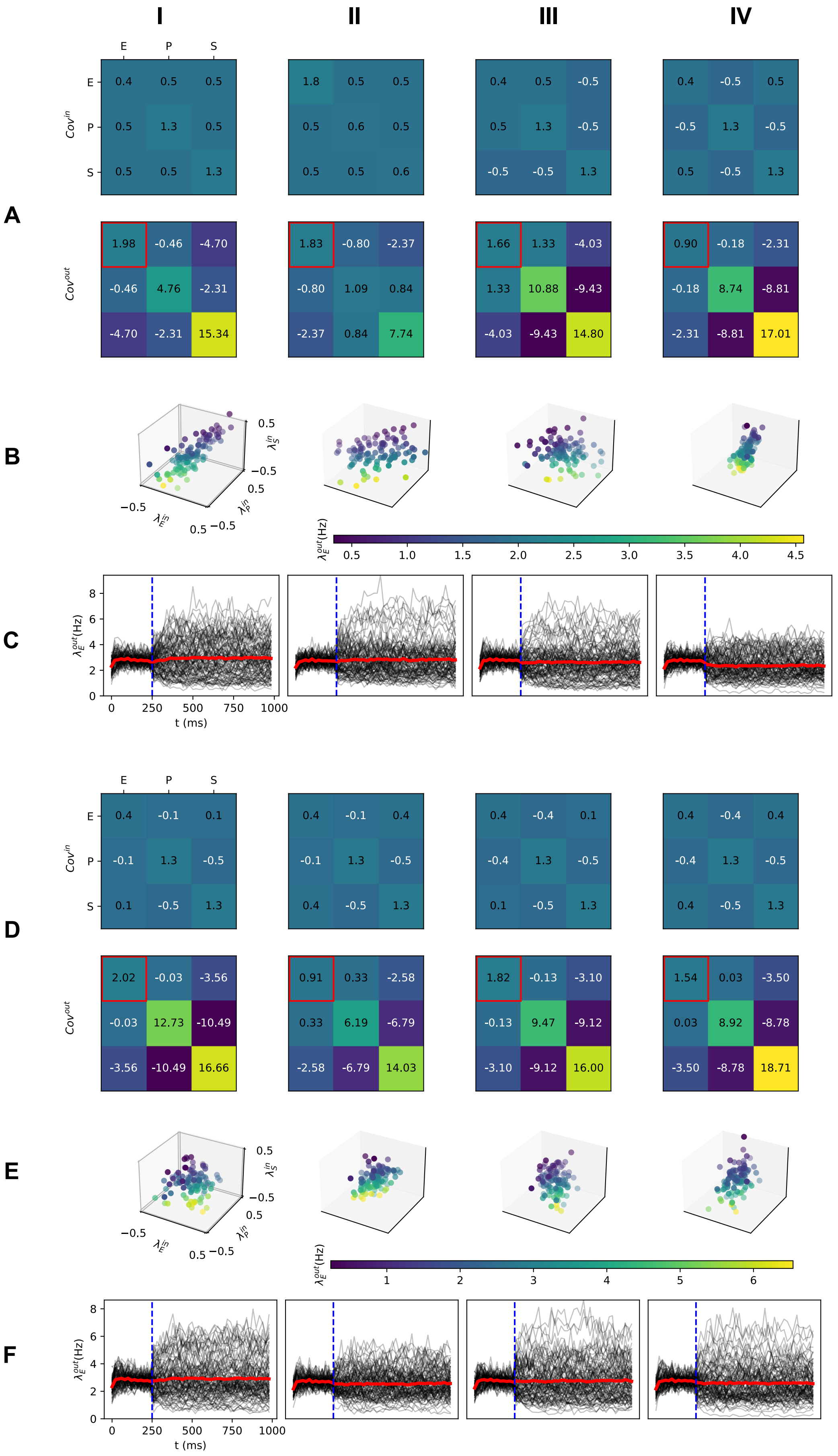

(**A top**) The input covariance matrix for sampling inputs for each trial (**B**). From **I** to **II**, the trial-by-trial input balance between E and SST/PV populations were increased. In column **III** and **IV**, negative variance in $Cov^{in}$ denotes the negative slope of input point clouds as shown in Figure 2F. The network operates in a state corresponding to the middle row in Figure 3D. (**A bottom**) Output covariance matrix across 100 trials where inputs for each trial were sampled from the covariance matrix in **A top**. The output variance of the excitatory population is marked with a red square. (**B**) Trial-by-trial input point clouds sampled from given covariance matrix as columns **I** to **IV** in **A top** (axes are normalized to the maximal value in Figure 1D.) **C** PSTH of excitatory population response. Black lines: individual trial. Red line: average response over 100 trials. Stimulus is given at 250ms, and output covariance matrix in **A bottom** is calculated for the last 500ms.(**D top**) The input covariance matrix for sampling input for each trial. From **I** to **II** (**III**), the trial-by-trial input covariance between E and SST (PV) populations were increased. In column **IV**, both covariances were increased. (**D bottom**) Output covariance matrix across 100 trials where inputs for each trial were sampled from the covariance matrix in **D top**. The output variance of the excitatory population is marked with a red square. (**E**) Trial-by-trial input point clouds sampled from given covariance matrix as columns **I** to **IV** in **D top** (normalized range). (**F**) PSTH of excitatory population response. Black lines: individual trial. Red line: average response over 100 trials. Stimulus is given at 250ms, and the output covariance matrix in **D bottom** is calculated for the last 500ms.

The trend is consistent with Figure 3C middle row. Exact values are slightly different due to linear interpolation of neuron transfer-function and limited sampling size for simulations while the actual underlying iso-firing rate surfaces are nonlinear Figure 1D right.
